# QUANTIFYING SENSITIVE DEPENDENCE OF INITIAL CONDITION USING STRUCTURAL EQUATION MODELING

**DOI:** 10.1093/geroni/igz038.1382

**Published:** 2019-11-08

**Authors:** Robert Moulder, Steve Boker

**Affiliations:** University of Virginia, Charlottesville, Virginia, United States

## Abstract

Human systems display sensitive dependence of initial condition. That is, even though two individuals may be similar in most regards, small differences between these individuals may have far reaching consequences later in life. In dynamical systems analysis, this sort of behavior is quantified with maximum Lyapunov exponents. These exponents quantify the degree to which small differences in initial condition between two systems affect trajectories of these systems later in time. Current methods for estimating maximum Lyapunov exponents are sensitive to noise and this sensitivity leads to estimation errors when researchers attempt to estimate these exponents on data obtained from human participants. Additionally, most current methods only allow for maximum Lyapunov exponent estimation using univariate time series. In this presentation, we present a method for using structural equation modeling for estimating latent maximum Lyapunov exponents from noisy multivariate time series and discuss applications of this method for analyzing human generated data.

